# Isolation and Characterization of Two Lytic Bacteriophages Infecting a Multi-Drug Resistant *Salmonella* Typhimurium and Their Efficacy to Combat Salmonellosis in Ready-to-Use Foods

**DOI:** 10.3390/microorganisms9020423

**Published:** 2021-02-18

**Authors:** Ahmed Esmael, Ehab Azab, Adil A. Gobouri, Mohamed A. Nasr-Eldin, Mahmoud M. A. Moustafa, Shereen A. Mohamed, Omnia A. M. Badr, Alzahraa M. Abdelatty

**Affiliations:** 1 Botany and Microbiology Department, Faculty of Science, Benha University, Qalubiya Governorate 13511, Egypt; nasreldeen.m@gmail.com; 2 Department of Biotechnology, College of Science, Taif University, P.O. Box 11099, Taif 21944, Saudi Arabia; e.azab@tu.edu.sa; 3 Department of Chemistry, College of Science, Taif University, P.O. Box 11099, Taif 21944, Saudi Arabia; a.gobouri@tu.edu.sa; 4 Department of Genetics and Genetic Engineering, Faculty of Agriculture, Benha University, Qalubiya Governorate 13736, Egypt; mahmoud.mustafa@fagr.bu.edu.eg (M.M.A.M.); shereen.mustafa@fagr.bu.edu.eg (S.A.M.); omnia.badr@fagr.bu.edu.eg (O.A.M.B.); 5 Department of Nutrition and Clinical Nutrition, Faculty of Veterinary Medicine, Cairo University, Giza 12613, Egypt

**Keywords:** *Salmonella* Typhimurium, Foodborne salmonellosis, Bacteriophage, Biocontrol

## Abstract

Foodborne salmonellosis is a global threat to public health. In the current study, we describe the isolation and characterization of two broad-spectrum, lytic *Salmonella* phages: SPHG1 and SPHG3 infecting a multidrug-resistant *Salmonella* Typhimurium EG.SmT3. Electron microscopy and whole genome analysis identified SPHG1 as a Myovirus, while SPHG3 as a new member of the genus “*Kuttervirus*” within the family *Ackermannviridae*. SPHG1 and SPHG3 had a lysis time of 60 min. with burst sizes of 104 and 138 PFU/cell, respectively. The two phages were robust at variable temperatures and pH ranges that match the corresponding values of most of the food storage and processing conditions. A phage cocktail containing the two phages was stable in the tested food articles for up to 48 h. The application of the phage cocktail at MOIs of 1000 or 100 resulted in a significant reduction in the viable count of *S.* Typhimurium by 4.2 log_10_/sample in milk, water, and on chicken breast. Additionally, the phage cocktail showed a prospective ability to eradicate and reduce the biofilm that formed by *S.* Typhimurium EG.SmT3. A phage cocktail of SPHG1 and SPHG3 is considered as a promising candidate as a biocontrol agent against foodborne salmonellosis due to its broad host ranges, highly lytic activities, and the absence of any virulence or lysogeny-related genes in their genomes.

## 1. Introduction

Foodborne infections that are caused by non-typhoidal *Salmonella* denote a considerable public health threat worldwide [1]. *Salmonella* is a Gram-negative bacilliform bacterium, which belongs to the family of *Enterobacteriaceae*. It is responsible for one of the most common food-borne illnesses, known as Salmonellosis. Previously, salmonellosis outbreaks were associated with the consumption of contaminated food products, and beef, pork, poultry, and dairy products were the major causative agents. Salmonellosis symptoms are abdominal cramps, fever, vomiting, inflammatory diarrhea, and nausea occurring within 12–72 h of infection and last from 2–7 days. Severe invasive *Salmonella* infections, such as bacteremia and septicemia, often arise in immunocompromised people, leading to hospitalization and death [2].

Globally, it is estimated that *Salmonella* spp. outbreaks are annually responsible for nearly about 85% (80.3 million cases) of diseases that are associated with foodborne diseases (93.8 million cases), resulting in over 100,000 deaths [3]. In 2007, the Unites States Department of Agriculture Economic Research Services (USDA-ERS) estimated an economic losses of US $2.5 million, due to 1.4 million cases of salmonellosis [4]. Throughout the world, different types of *Salmonella* serotypes have been associated with foodborne illness, including *S*. Enteritidis and *S*. Typhimurium [5].

*Salmonella* species are frequently depicted as environmental persisters [6,7], and has the ability to form surface-associated complex communities that are known as biofilms on food [8]. *Salmonella* biofilm may serve as bacterial reservoir for recurrent bacterial contamination in a food processing facility, and cause many food-borne outbreaks [9]. A consumption of *Salmonella* biofilm-contaminated chicken resulted in *Salmonella* outbreaks, with about 2138 cases of infection [9]. According to the Centers for Disease Control and prevention, biofilms are responsible for about 80% of most bacterial disease [10]. Microbial biofilms pose a serious threat to food industry, as they are difficult to inactivate or eradicate, owing to their inherent resistance to traditional physical and antimicrobial treatments.

Conventional intervention strategies to control and eliminate *Salmonella* serovars and biofilms in food products are predominantly carried out while using certain biocides, chemical sanitizers, heat treatments, and other special preservatives [2,11,12,13]. Although those strategies are effective, they develop undesirable impacts on the quality of food products. Of these, chemical residues can alter the taste, texture, and aroma [14], and some preservatives can cause side effects, such as asthma, rashes, allergies, and hemorrhagic diarrhea [15,16]. In addition, certain essential vitamins may be destroyed following heat treatment, which renders the food to be less nutritive [17,18,19].

Because most conventional methods showed undesirable outcomes, as well as having limited impact on *Salmonella* control, antibiotics were once considered as an effective method to reduce *Salmonella* in animals used for food production, however, later this was showing to lead to the emergence of multidrug-resistant *Salmonella* spp [20,21]. Antibiotics usage was restricted from Sweden in 1986, by the Danish Pig Production Committee in 1995 and the European Union in 1999 [22]. Subsequently, the application of antibiotics in food production has become largely discouraged. Notwithstanding many attempts to develop effective methodologies to eliminate microbial contamination, food safety is still a challenge because of the prevalence of antibiotic-resistant bacteria as well as food market globalization [23]. Thus, novel, or alternative, safe and effective agents are essential to solve the dilemma of food safety without altering the nutritive quality. As a novel strategy, bacteriophages have emerged as a promising natural approach for food safety and preservation [24,25,26,27].

Bacteriophages or viruses of bacteria are ubiquitous [28,29], with approximate titers of 10^31^ phage particles on the planet [30]. As a novel strategy, lytic (virulent) phages are promising candidates as antimicrobial agents in the food industry, as they replicate exponentially in their susceptible hosts, regardless of any multidrug resistance [26,27,31,32]. Phage applications are safe, because they are environmentally friendly; moreover, phages can easily be detected from healthy humans, animals, and foods with to date no reported phage infection of humans [33,34,35,36,37]. Phages have been applied to combat salmonellosis in different foods including chicken [38,39,40], raw and cooked beef [41], pig skin [42], sprout seeds, fresh-cut fruits [43], as well as in cheese production [44]. Currently, some phage products have been granted Generally Recognized as Safe (GRAS) status, by the FDA, for example, SalmoFresh™, ListShield™, and PhageGuard S™ are commercially available products for food applications [27].

To be applied as biocontrol agents in the food industry, phages should have certain features, as a broad host range, persist the food processing environment, and do not have any pathogenic or allergic-associated properties [45,46]. Moreover, temperate, or lysogenic phages are disqualified as biocontrol agents, because they are less effective and can integrate into their hosts, which facilitates the transferring of antibiotic-resistant genes or virulence genes to their bacterial host that increase the potential of generating pathogenic strains [27,47].

Prior to this study, five *Salmonella enterica* serovars were isolated from a poultry farm with a history of diarrhea in Benha city, Qalubiya governorate, Egypt. Antibiotic sensitivity testing of the isolated *Salmonella* spp identified a multidrug-resistant isolate (*Salmonella* Typhimurium strain EG.SmT3). In this study, we depict the isolation and characterization of *Salmonella* phages from Egypt against *S.* Typhimurium EG.SmT3 with the aim of developing biocontrol agents to combat food-borne salmonellosis in diverse food samples. Two strictly lytic phages, SPHG1 and SPHG3, were selected due to their high lytic activity and broad host ranges for further investigation and genome sequencing.

## 2. Materials and Methods

### 2.1. Bacterial Strains and Growth Conditions

The current study was performed on a multi-drug resistant *Salmonella enterica* serovar (*Salmonella* Typhimurium strain EG.SmT3, GenBank Acc. No. MW310702). All of the Bacteria in this study were kindly taken from the culture collection of the Microbiology lab, Botany and Microbiology Dept., Faculty of Science, Benha University, Egypt.

The bacteria were stored at −80 °C in Brain-Heart-Infusion broth that was supplemented with 20% (*v/v*) glycerol. Before every experiment, fresh overnight cultures were prepared by inoculating a single colony into 10 mL tryptic soy broth (TSB, Difco, Detroit, MI, USA) and incubating for 16 h at 37 °C with shaking at 200 rpm.

### 2.2. Mitomycin C induction to Identify Prophage-free Salmonella

Bacteriophages isolation, propagation, and all of the following experiments were performed using the antibiotic-resistant strain *Salmonella* enterica subsp. enterica serovar Typhimurium EG.SmT3. Prior to the isolation of lytic bacteriophages, prophage-free (non-lysogenic) *S*. Typhimurium EG.SmT3 was identified using mitomycin C induction protocol [48]. Briefly, 5 mL of a mid-log phase *Salmonella* culture grown in TSB media was subjected to a final concentration of 0.2 µg/mL of mitomycin C (Sigma-Aldrich, St. Louis, MO, USA). The bacterial growth was monitored by measuring the absorbance at OD_600nm_. Regularly, 500 µL aliquots of the sample was collected, centrifuged to remove residual bacteria, and assessed for prophage induction. Briefly, prophage induction was detected by spotting a 10 µL from each supernatant onto a lawn of *S*. Typhimurium EG.SmT3 and then incubated for 24 h at 37 °C [49,50].

### 2.3. Bacteriophages Enrichment and Isolation

Different environmental samples were collected from Benha city, Qalubiya governorate, Egypt, including a wastewater treatment plant, an agricultural farm ditch, and chicken feces, as described previously [51]. Solid particles were removed from the collected water samples by centrifugation at 10,000× *g* for 10 min., cellular microorganisms in the samples were excluded by membrane filtration using 0.22 µm membrane filters (Mixed Cellulose Ester, MF-Millipore, Burlington, MA, USA). Chicken feces were dissolved in 10 mL tryptic soy broth (TSB), and then processed in the same way as the environmental water samples.

The enrichment of phages and isolation were performed, as described previously [52]. Briefly, 5 mL of a 0.22 µm-filtered sample was mixed with 5 mL double-strength TSB medium and 100 µL of *S.* Typhimurium EG.SmT3 and then incubated for 24 h at 37 °C with shaking at 200 rpm. The enriched tubes were then centrifuged at 5000× *g* for 10 min., and the supernatants were filtered using a 0.22 µm membrane filters. Phages activity was detected by spotting a 10 µl from each supernatant onto a lawn of the indicator *Salmonella* strain and incubated for 24 h at 37 °C [49,50]. The plates were examined for the presence of lysis zones, and any lysis zones were cut from the TSA plates using sterile pipette tips and then transferred into separate clean tubes containing 200 µL salt-magnesium (SM) buffer (0.05 M Tris-HCl; 0.1 M NaCl; and, 0.01 M MgSO_4_; pH 7.5) and stored overnight at room temperature to allow for the phage particles to diffuse into the SM buffer.

### 2.4. Bacteriophages Purification and Propagation

The purification of the isolated phages was done using the double agar overlay method [53]. Individual plaques with different morphologies and sizes were picked from the overlay plates using sterile toothpicks, separately resuspended in 200 µL SM buffer and held overnight at room temperature. The resuspended plaques were plated using the double agar plate and the isolation of single plaques was repeated three successive times.

Propagation of the purified phages was performed, as previously described [54,55]. Double agar overlay method was used with multiple phage dilutions, plates with full lysis were washed with 5 mL of SM buffer at 4 °C overnight, shaking at 90 rpm. The surface liquid was removed, vortexed, and centrifuged at 5000× *g* for 15 min. at 4 °C. The supernatant was filtered using a 0.22 µm membrane filters (Millipore, Ireland). Highly purified phage particles were obtained, as described previously [54,55]. Phage titer (PFU/mL) was determined using the double agar overlay method [53]. All of the isolated and purified phages were stored in SM buffer at 4 °C for further analysis.

### 2.5. Virulence and Lytic Activity

The virulence of the isolated phages against *S.* Typhimurium EG.SmT3 was investigated, as described previously [49], in a 96-well microplate and a multiplicity of infection of 1 by measuring the optical density (OD_600nm_) for 6 h post-infection. Each test group contained a mixture of equal volumes (100 µL each) of exponential-phase *S.* Typhimurium EG.SmT3 cultures (7 log_10_ CFU/mL) and diluted phage lysates (7 log_10_ PFU/mL). The negative control consisted of a mixture of equal volumes of *S.* Typhimurium EG.SmT3 and TSB. All of the plates were incubated at 37 °C with shaking at 120 rpm for 6 h and optical densities were measured at 600 nm while using a microplate reader (680 XR reader, Bio-Rad, Hercules, CA, USA). Phages with high lytic activities were selected for further experiments.

### 2.6. Characterization of the Selected Phages

#### 2.6.1. Determination of Host Range by Efficiency of Plating (EOP)

The host range for the two selected phages (SPHG1 and SPHG3), as well as a cocktail of those two phages (with a ratio of 1:1), was determined against a collection of fifteen *Salmonella* strains and a cohort of six non-*Salmonella* strains (Table S1). To determine the host range, efficiency of plating (EOP) was performed, as previously described with some modifications [56,57]. Each isolated phage was serially ten-fold diluted and tested, in triplicates, on the TSA bacterial lawn plates and the incubated at 37 °C for 16–18 h. The number of plaques forming units (PFUs) was counted, and the efficiency of plating was calculated, as follows:

EOP = average of PFUs on test bacteria/average of PFUs on the host bacteria. EOP was classified as high efficiency, EOP 0.5 to 1.0; moderate efficiency, EOP 0.2 to <0.5; low efficiency, 0.0001 to <0.2; and, inefficient < 0.001 [56,57].

#### 2.6.2. Transmission Electron Microscopy (TEM)

Ten microliters of each highly purified phage (~10^12^ PFU/mL) were fixed onto copper grids (Electron Microscopy Sciences) that were supported by carbon-coated Formvar film [58]. Phages were then negatively stained with 2% (*w*/*v*) aqueous phosphate tungsten acid, pH 7.2 for 1 min. and then air-dried for 1 h at room temperature. A JEOL JEM-2100 transmission electron microscope was used for acquiring the phage particle images at the Electron Microscope Facility, Al-Mansoura University, Egypt.

#### 2.6.3. One-Step Growth Curve

Phages growth kinetics and burst size were determined, as described previously [49]. A known number of *S.* Typhimurium EG.SmT3 cells were infected with phages individually at a MOI of 1. After 5 min. of adsorption at room temperature, the infected bacteria were centrifuged at 5000 *× g* for 5 min. and the supernatant was discarded to remove free-unbound phages. The phage-bacteria pellet was then washed twice and resuspended in 10 mL of TSB and then incubated at 37 °C with continuous shaking. At appropriate times, phage titers were enumerated using plaque assay [53]. The experiment was independently repeated three times, mean burst size (plaque-forming units per cell) at different times were calculated and plotted against time to determine the latent period and burst size.

#### 2.6.4. Genomic Analysis of the Isolated Phages

Genomic DNAs of the isolated phages (SPHG1 and SPHG3) were extracted and purified, as described previously [59]. Nucleotide sequencing was performed employing the Illumina HiSeq 4000 platform (Illumina, San Diego, CA, USA) by means of a pair-end library with a 150 bp read length. The reads were assembled using MicrobeTrakr plus (v 0.9.1) software (ShangHai, China) resulting in a unique contig for each phage. Open-reading frames were detected using NCBI ORF finder search server. Functional annotation of the putative coding sequences (CDSs) was identified using the BLASTp search against the NCBI non-redundant database. The annotated genes for each phage were then manually curated and listed in Supplementary Table S4. Genes encoding tRNAs were detected using tRNAscan-SE v.1.3.1 [60]. The Genomic circular map of each phage was prepared using CGView [61]. The annotated complete genome of phages SPHG1 and SPHG3 have been deposited in the GenBank database under accession numbers of MW413353.1 and MW388005.1, respectively.

#### 2.6.5. Thermal and pH Stability

Thermal and pH-stability testing of the selected phages was performed, as described previously [49]. For thermal-stability, 900 µL of pre-heated sterile 2 × TSB medium were mixed with 100 µL of phage lysates (10 log_10_ PFU/mL). The tubes were incubated in a water bath ranging from 30 °C−80 °C for either 30 min. or 60 min., respectively. For pH-stability assessment, phage lysates (10 log_10_ PFU/mL) were diluted in 2 × TSB tubes at a pH range of 2–13 and then incubated 24 h at 37 °C. Each temperature and pH treatment was performed in triplicate, and the average of triplicate counts was calculated. Thermal and pH tolerance rates were calculated by determining phage titers using the double-layer agar plate, as follows:

Phage thermal/pH stability (%) = (Remaining phage titers following the treatment / Phage titer before treatment) × 100%(1)

### 2.7. Biological Control of Salmonella in Food Using Phage Cocktail

#### 2.7.1. Development of Phage Cocktail

A phage cocktail was developed by mixing the SPHG1 and SPHG3 phages with a ratio of 1:1, each phage at a titer of 10 log_10_ PFU/mL. The cocktail was later diluted in sterile SM buffer to reach the objective concentration.

#### 2.7.2. Stability of Phage Cocktail in Food

Pasteurized milk and boneless chicken breasts were purchased from local stores and the water used is sterile faucet water from Benha city, Egypt. Prior to the experiment, chicken breast slices (1 cm^2^) were washed thoroughly with sterile water to reduce the background bacteria.

The stability of phage cocktail in milk, water, and on chicken breasts at different temperatures (4 °C and 25 °C) was evaluated for two days, as described previously [62]. Briefly, phage cocktail (8 log_10_ PFU/mL) was added into sterile milk and water, phage cocktail was spotted on the surface of chicken breast at a final titer of 8.3 log_10_ PFU/cm. The infected food samples were then incubated at 4 °C and 25 °C for 2 days. At appropriate times, phage titers were enumerated using the plaque assay [53].

#### 2.7.3. Recovered Bacterial Load from Treated Foods

*Salmonella* biocontrol experiments using a phage cocktail were performed, as described previously [62]. Briefly, 10 µL of *S*. Typhimurium EG.SmT3 (4 log_10_ CFU/mL) was individually added to milk and water, and then the phage cocktail was added at a MOI of 100 (6 log_10_ PFU/mL) or a MOI of 1000 (7 log_10_ PFU/mL). To assay on chicken breast, each 1 cm^2^ slice of the chicken breasts was spotted with 10 µL of *S*. Typhimurium EG.SmT3 (4 log_10_ CFU/mL), dried for 30 min., and finally, the phage cocktail was added at MOIs of 100 and Phages-free SM buffer was added to the food samples in the control group. Samples were incubated at either 4 °C or 25 °C for 48 h. The aliquots were collected at a designated time post-infection to determine the recoverable bacteria counts.

### 2.8. Effect of Phage Cocktail against Biofilm of Salmonella Typhimurium EG.SmT3

The effectiveness of the phage cocktail to reduce biofilm of *S*. Typhimurium EG.SmT3 was quantitatively determined according to the previously described colorimetric method [62,63], with some modifications. In each well of the 96-well microplate, *S*. Typhimurium EG.SmT3 (final concentration of 4 log_10_ CFU/mL) was inoculated into LB medium without NaCl, and then the plate was incubated under static condition at 30 °C for three days, medium was renewed every 24 h. Subsequently the bacterial wells were challenged with the phage cocktail at a final titer of 7 log_10_ and 8 log_10_ PFU/mL, for negative controls phosphate buffer saline (PBS) was used instead of the phage cocktail. Plates were further incubated under static condition at 30 °C for 24 h, and then the wells were rinsed five times with PBS and allowed to air-dry. The air-dried plates were then treated with 98% methanol for 10 min., the methanol was removed, and then plates were air dried again. The plates were then stained with 1% crystal violet for 45 min. and eluted using 33% acetic acid. Optical densities were measured at 600 nm using a microplate reader (680 XR reader, Bio-Rad). Biofilm reduction percentages were calculated, as follows:

Biofilm reduction (%) = [(Average OD_600 nm_ of the control − Average OD_600 nm_ of phage-treated wells)/Average OD_600 nm_ of the control] × 100% (2)

## 3. Results

### 3.1. Bacteriophages Isolation, Selection and Lytic Activity

To avoid mixed (lytic and lysogenic) phage populations within the individual plaques because of probable prophage induction, *S.* Typhimurium EG.SmT3 was checked for lysogeny by inducing potential prophages using mitomycin C. *S.* Typhimurium EG.SmT3 was found to be negative for prophage induction by mitomycin C, which suggested that it is suitable as a phage isolation host.

A total of five phages were successfully isolated, purified, and propagated using *S.* Typhimurium EG.SmT3 as a target host for isolation and enrichment. Phages SPHG1 and SPHG3 were isolated from Benha wastewater treatment plant; phages SPHG2, SPHG4, and SPHG5 were isolated from chicken manure. The five isolated phages showed discrete differences in plaque size and turbidity shape.

**Figure 1:**
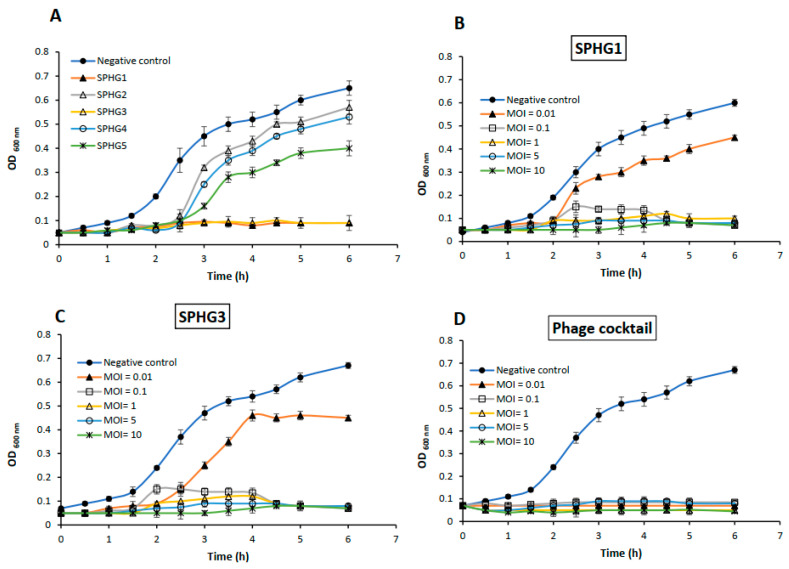
(**A**) Lytic ability of the five isolated phages on *S.* Typhimurium, EG.SmT3 as a host at multiplicity of infection (MOI) of 1 in TSB broth, (**B**,**C**) Lytic ability of phages SPHG1 and SPHG3, respectively to lyse *S.* Typhimurium EG.SmT3 in TSB medium at different MOIs of 10, 5, 1, 0.1, and 0.01 at 37 °C, and (**D**) Lytic activity of a phage cocktail developed from phages SPHG1 and SPHGThe bacteria were challenged with the isolated phages at the designated MOI in 96-well microtiter plates and incubated at 37 °C. The bacterial growth was estimated by measuring optical densities (OD_600_ nm) up to 6 h post-infection. The data shown are the mean of three replicates and error bars show the deviation in the values.

To select the most effective phages, an examination of the lytic activity was conducted against *S.* Typhimurium EG.SmT3, as shown in Figure 1A. The results showed that all of the phages inhibited the host growth 2 h post-infection (p.i.); however, the SPHG2, SPHG4, and SPHG5 phages lost their activity 2.5 h p.i. Two phages (SPHG1 and SPHG3) were found to have high and retained lytic activities after prolonged incubation. These two phages (SPHG1 and SPHG3) and a cocktail of them were selected for further analysis in order to confirm their lytic activity at different multiplicities of infection (MOIs).

Phages SPHG1 and SPHG3 inhibited the growth of *S.* Typhimurium, EG.SmT3 6 h p.i. when used at 0.1, 1, and 5, as shown in Figure 1B,C; however, a lower MOI of 0.01, bacterial growth was seen 2 h p.i. Interestingly, the phage cocktail constantly inhibited the growth of *S.* Typhimurium EG.SmT3 with an extended inhibition for 6 h p.i. (Figure 1D) and it exhibited intense activity; therefore, it could be a potential candidate for the control of *Salmonella*.

### 3.2. Characterization of S. Typhimurium Selected Phages

#### 3.2.1. Host Range of Phages by Efficiency of Plating (EOP)

The host range pattern of phages chosen, as well as a cocktail of those phages, was determined by EOP (Table 1). The phage cocktail established the most significant and broadest spectrum of lytic activity as compared with single phages in our study. Phage cocktail lysed thirteen of the tested *Salmonella* strains (*n* = 15). Phage cocktail had a high efficiency (0.5–1.0) against all of the tested *S*. Typhimurium, but the EOP values were low to moderate (0–0.5) when other bacteria were challenged with the phage cocktail mix. For the single phage efficiency, SPHG3 showed the broadest spectrum of lytic activity against the assessed host strains. Neither the individual phages nor the phage cocktail mix broke the boundary of the genus and lysed other bacterial genera tested. which were *Staphylococcus aureus* and *Escherichia coli*.

#### 3.2.2. TEM Morphology and Growth-Kinetics of the Isolated Phages

**Figure 2:**
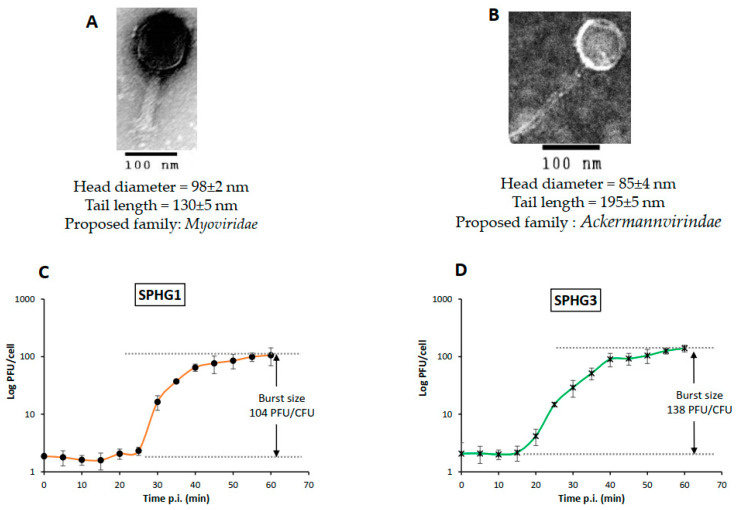
Morphology and Growth kinetics of phages SPHG1 and SPHG3: (**A**,**B**) Transmission Electron Microscope analysis of phages SPHG1 and SPHG3, respectively, head and tail measurements of each phage are represented below each micrograph, scale bar = 100 nm and (**C**,**D**) one-step growth curves of SPHG1 and SPHG3 phages on *S.* Typhimurium EG.SmT3. The data shown are the mean of three replicates and error bars show the deviation in the values.

The morphologies of the two selected phages were observed by TEM. All of the examined phages have isometric heads with contractile or non-contractile tails. Phage SPHG1 in Figure 2A belongs to the *Myoviridae* family, having a contractile tail. In contrast, phage SPHG3 has a long, flexible non-contractile tail, and, consequently, suspected to belong to the *Ackermannvirindae* family as displayed in Figure 2B. The respective diameters of the head and tail lengths were calculated and are shown in Figure 2. One-step growth curves were performed to characterize the two phages’ infection cycle, in order to determine burst sizes and latent periods (Figure 2C,D). The SPHG1 phage had a higher latent period (25 min) with smaller burst size (104 PFU/cell), as compared to SPHG3, which had a latent period of 15 min. with an average burst size of 138 PFU/cell.

#### 3.2.3. Analysis of Phage Genomes

The complete genomes of phages SPHG1 and SPHG3 have been sequenced, deposited in the GenBank database, and designed the accession numbers MW413353.1 and MW388005.1, respectively.

**Figure 3:**
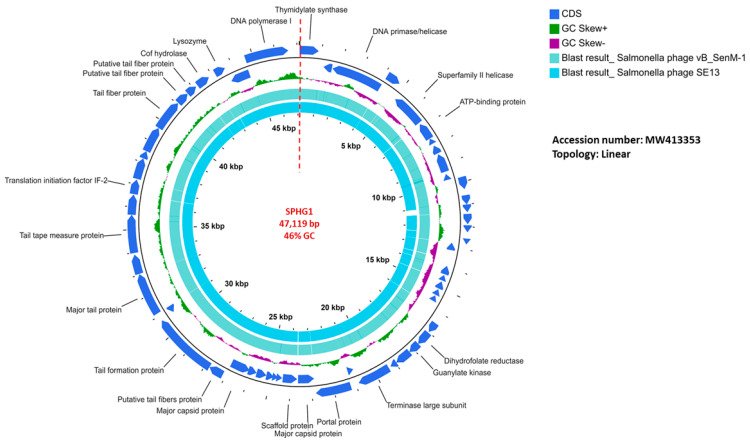
The SPHG1 genome organization represented as a circle. The 62 CDSs are represented as blue arrows showing the predicted genes transcribed clockwise (outer side) and counterclockwise (inner side) along the genome. The genome map is circle, but there is a break at the 12 o’clock position (at the dotted straight red line) because the phage genome is a linear molecule. CDSs with known functions are labelled along with their position; however, other non-labelled CDSs represent hypothetical proteins. The following cycle is the GC content of the genome. The inner two circles represent the BLASTn alignment of SPHG1 against *Salmonella* phage vB_SenM-1 and *Salmonella* phage SE13, respectively.

SPHG1 has a double-stranded DNA of 47,119 bp with an overall G + C content of 46% and it is presented in a linear topology in Figure 3. Blastn alignment against the previously sequenced phages (Table S2) shows that the genome of SPHG1 has a 97.59% degree of identity with the *Salmonella* phage VB_SenM-1 (GenBank Acc. No. MT012730.1). The determination of the open reading frames (ORFs) applying the standard genetic code and using ATG as initiation codon, identified sixty-two putative protein-coding genes and no tRNAs, among which twenty-two predicted proteins have known potential functions that are responsible for assigned functions: lysis, DNA packaging, structural genes, and DNA replication (Table S3). SPHG1 has 29 ORFs on the leading strand and 33 ORFs on the complementary strand. BLASTn analysis confirmed that SPHG1 is a member of the *Myoviridae* family, in the order *Caudovirales*.

**Figure 4:**
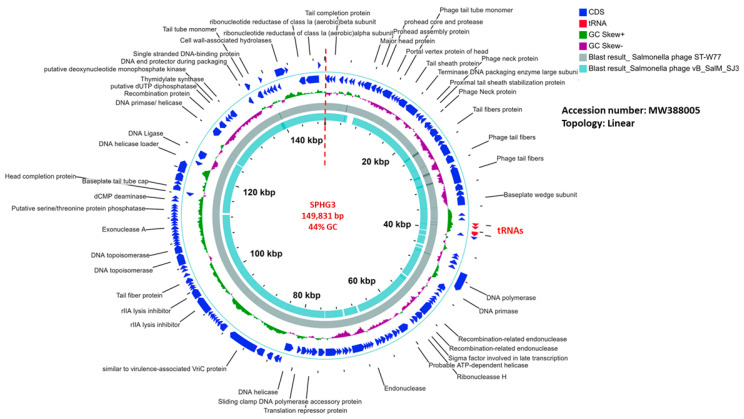
The SPHG3 genome organization represented as a circle. The 154 CDSs are represented as blue arrows showing the predicted genes transcribed clockwise (outer side) and counterclockwise (inner side) along the genome. Genes encoding tRNAs are represented as red arrows at about 3 o’clock in the genome. The genome map is circle, but there is a break at the 12 o’clock position (at the dotted straight red line) because the phage genome is a linear molecule. CDSs with known functions are labelled along with their position; however, other non-labelled CDSs represent hypothetical proteins. The following cycle is the GC content of the genome. The inner two circles represent the BLASTn alignment of SPHG3 against *Salmonella* phage ST-W77 and *Salmonella* phage vB_SalM_SJ3, respectively.

Sequencing of the SPHG3 genome established that it was 149,831 bp long with a G + C content of 44%; Figure 4 displays the linear map of the genome organization. The SPHG3 is predicted to encode 149 CDSs and 5 tRNA genes at the (Table S5). Within the total ORFs, SPHG3 has 52 ORFs on the leading strand, and 97 ORFs on the complementary strand. BLASTp analysis identified 64 predicted proteins with putatively known functions. Among which, they could classify as structural proteins, DNA replication/transcription/repair proteins, cell lysis proteins, nucleotide metabolism proteins, and DNA packaging proteins. BLAST search (Table S4) identified a 99% nucleotide similarity of the SPHG3 genome with the previously sequenced *Salmonella* phage ST-W77 (GenBank Acc. No. NC_049378.1). SPHG3 is classified as a member of the genus *Kuttervirus*, subfamily *Cvivirinae*, family *Ackermannvirindae*, order *Caudovirales*.

The BLASTp search indicated that the SPHG1 and SPHG3 genome do not encode any genes that are related to lysogeny (virulence factors, toxins, antimicrobial-resistant genes, repressors, transposases, or integrase encoding genes), which suggests that the SPHG1 and SPHG3 are virulent and are safe for the application.

#### 3.2.4. pH and Thermal Stability

**Figure 5:**
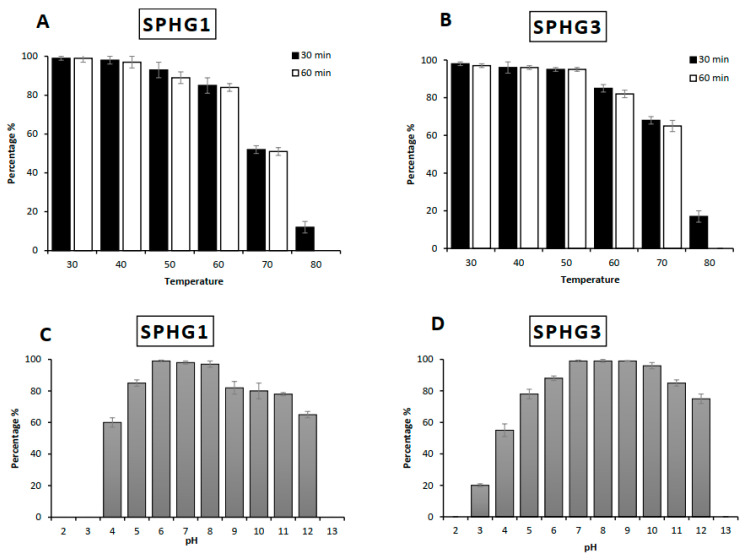
Thermal and pH stability test of phages SPHG1 and SPHG3. (**A**,**B**) Thermal tolerance of SPHG1 and SPHG3 phages respectively, and (**C**,**D**) pH stability of SPHG1 and SPHG3 phages, respectively. Temperature experiments were performed for 30 min., and 60 min. at pH 7. pH experiments were performed for 24 h at 37 °C. The data showed the percentages of the remaining phages after each treatment, as normalized from the control. The data reported are means of three independent trials and error bars show the deviation in the values.

The thermal and pH stability patterns of phages SPHG1 and SPHG3 were determined based on residual phage titers after incubation under various conditions, as presented in Figure 5. Phages exhibited a high degree of thermal stability from 30 °C to 70 °C, as in Figure 5A,B. However, after heating at 80 °C for 30 min., phage titers decreased by 80% for both phages, and no viable phages were detected after heating at 80 °C for 60 min. Regarding pH stability, SPHG1 and SPHG3 phages were found to be resistant to inactivation at a pH range of 4–12 after 24 h with most survival seen at pH values of 7, 8, and 9, as illustrated in Figure 5C,D. Although no phages were detected at pH < 4 or >12 for the SPHG1 phage, the SPHG3 phage retained some activity at pH 3.0.

### 3.3. Application of Phage Cocktail to Control Foodborne Multi-Drug Resistant S. Typhimurium

**Figure 6:**
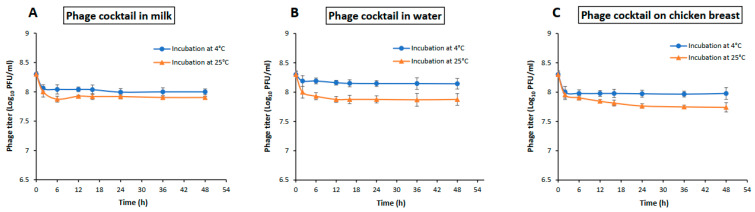
Stability of *Salmonella* phage cocktail in different food articles. (**A**) Stability in milk, (**B**) stability in water, and (**C**) stability on chicken breast. Phage cocktail titer of 8.3 log_10_ PFU/mL was mixed with each food sample and incubated at either 4 °C or 25 °C for 48 h. The values represent mean with a standard deviation of three replicates of each time point.

A phage cocktail composed of 1:1 mixture of phage SPHG1 and SPHG3 was evaluated for the biological control of experimentally *S.* Typhimurium EG.SmT3 contaminated milk, water, and chicken breasts. The stability tests were determined at two different temperatures representative of storage temperature of most food articles (at 4 °C) and the temperature at which the food is being processed or consumed (at 25 °C). The results in Figure 6 indicated that the phage cocktail remained stable in the tested food matrices.

**Figure 7:**
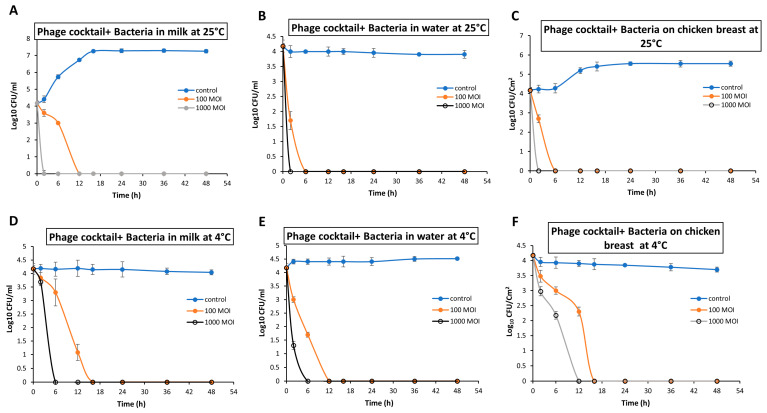
Biocontrol of S. Typhimurium EG.SmT3 using *Salmonella* phage cocktail at different food matrices. (**A**,**D**) Application in milk, (**B**,**E**) application in water, and (**C**,**F**) application on chicken breasts. Phage cocktail at to different MOIs (100 and 1000) were added separately to each food object and incubated either at 4 °C or 25 °C for 48 h, the un-infected control consisted of phage-free bacteria with SM buffer added. Values represent mean CFU/mL with a standard deviation of three replicates of each time point.

The food samples were artificially contaminated with *S.* Typhimurium EG.SmT3 at a final concentration of 4 log_10_ CFU/mL at either 4 °C or 25 °C. In the milk biocontrol assay, the viable count of *S.* Typhimurium EG.SmT3 was reduced below the detection limit (<1 CFU/100 µL) after 2 h and 12 h at 25 °C using MOIs of 1000 and 100, respectively (Figure 7A). While at 4 °C, there was a complete elimination of *Salmonella* after 6 h and 16 h using MOIs of 1000 and 100, respectively (Figure 7D). The effectiveness of the phage cocktail to inhibit the degree of experimentally contaminated *Salmonella* in water was also investigated (Figure 7B,E). No viable bacterial counts were detected in water after 2 h and 6 h at 25°C upon adding the phage cocktail at an MOI of 1000 and 100, respectively. While at 4 °C, the bacterial count completely declined after 6 h and 12 h using an MOI of 1000 and 100, respectively.

In Chicken breasts experiments, at 25 °C, the *Salmonella* counts were reduced completely after 2 h and 6 h upon application of the phage cocktail at an MOI of 1000 and 100, respectively (Figure 7C). However, the time that is required to achieve complete bacterial lysis increased at 4 °C to 12 h and 16 h when MOIs of 1000 and 100 were used, respectively (Figure 7F).

### 3.4. Effect of Phage Cocktail against Biofilm of S. Typhimurium

**Figure 8:**
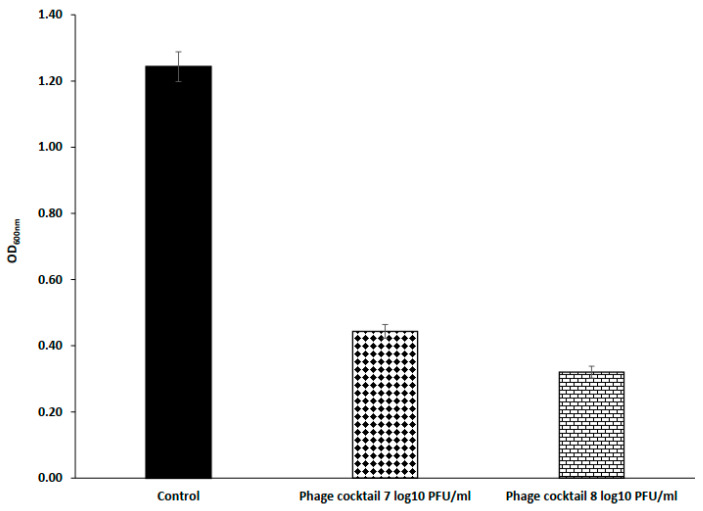
Effect of a phage cocktail on 72-h-old biofilm of *S.* Typhimurium EG.SmT3 in 96-well microplate. The values represent mean biofilm reduction with standard deviation of three replicates.

The effectiveness of phage cocktail against biofilm of *S.* Typhimurium EG.SmT3 in 96-well microplate was evaluated at 30 °C using titers of 7 log_10_ PFU/mL and 8 log_10_ PFU/mL for 24 h (Figure 8). Biofilm removal activities of 64.34% and 74.26% were respectively detected when phage cocktail was applied to a final titer of 7 log_10_ PFU/mL and 8 log_10_ PFU/mL.

## 4. Discussion

Guaranteeing the microbiological safety of food is an increasing concern among producers and consumers at all levels of the food production chain. The situation is becoming more complex with the emergence of foodborne multi-drug resistant bacteria [20,21]. Foodborne salmonellosis is the second most reported bacterial zoonosis. The European Union (EU) categorized *Salmonella* to be a major cause of foodborne illness [64].

MDR *Salmonella* serovars were previously isolated from Egypt [65,66,67,68,69]. Traditional intervention methods for combating *Salmonella* have not been able to solve the dilemma of food quality and food safety [14,15,16,17]. Bacteriophages have emerged as a promising alternative to chemical antimicrobial agents among the numerous attempts to improve food safety currently under exploration [24,25,26,27].

Previously, three lytic bacteriophages infecting MDR *S.* Typhimurium have been isolated from sewage in Egypt [69]. In the current study, five bacteriophages were isolated from different environmental samples targeting the MDR *S*. Typhimurium EG.SmT3. Two out of the five isolated phages were selected for further phage biocontrol experiments based on their inhibition activity. The data revealed that, the higher the MOIs over the bacterial concentration, the greater the relevance to the outcome of the treatment. The two phages, SPHG1 and SPHG3, as well as a cocktail of these two phages, exhibited broad host range activity and high efficiency to inactivate the tested *Salmonella* in the current study.

Host range analysis identified that SPHG3 had a broader host range and it was able to infect 86% of the tested *Salmonella* serovars, although SPHG1 only infected 53% of the strains tested, respectively. This difference in the susceptibility pattern of the isolated phages may result from unsuccessful infection, non-specific binding receptors, modification of the restriction endonuclease system [70,71,72], and the formation of bacterial insensitive mutants (BIM) [73]. Here, a cocktail mix of the two isolated phages was used. Phage cocktails overcome the limitation of phages with narrow host range [26]. Moreover, different phages recognize different receptor sites on the host cell wall; subsequently, this will delay or even prevent the development of host resistance [74,75,76].

Whole genome sequencing of both SPHG1 and SPHG3 was performed to screen for integrase genes as well as well virulence-associated genes. SPHG1 and SPHG3 have double-stranded DNA genomes of 47,119 bp and 149,831 bp long, respectively. Remarkably, SPHG1 and SPHG3 did not encode any integrases, virulence associated-factors, or antimicrobial-resistance genes. For biocontrol applications in food, strictly lytic phages are used to avoid potential threats (e.g., the transduction of virulence factors) that are associated with the lysogenic (temperate) phages [45,77,78]. Genome analysis identified SPHG1 and SPHG3 as virulent phages in the *Myoviridae* and *Ackermannvirindae* families, respectively. Previous literatures suggested that phages belonging to these families have the potential to be used as biocontrol agents against different *Salmonella* [59,69,79,80,81]. As such it is important to note that a mix of phages with distinct biological and genetic features can improve the effectiveness of a phage cocktail [82].

Phage applications in the food industry are of success based on the stability of the selected phage/s on different food matrices. Previously, insignificant losses were observed in phage titers in various food matrices [81,83], and the current results (Figure 6) showed that the phage cocktail was effectivity stable, with only small losses being seen after exposure for up to two days in milk, water, and on chicken breast. A recent study also reported the stability of a *Salmonella* phage cocktail in milk and on chicken breast [62]. Phages were reported to be more active at higher MOIs in a very short time [39,62,84]. In the current study, an MOI of 1 was considered the critical threshold level for the application of the isolated *Salmonella* phage cocktail. It has been established that, the higher the MOI value, the greater the reduction rate [85,86]. Moreover, using a high MOI of the applied phages has been shown to be effective, as it favors rapid attachment to the host cell wall receptors and it can result in the degradation of the cell wall without multiplication [42,62,87] via the “lysis from without” phenomena [88].

The results of the current study indicated a reduction in the growth of the MDR S. Typhimurium EG.SmT3 in artificially contaminated food matrices by the phage cocktail for up to 48 h as compared to the non-challenged samples, especially at 25 °C. The phage cocktail effectively reduced the initial count of *Salmonella* (~4.2 Log_10_ unit) below the detection limit (<1 CFU/100 µL) either at 4 °C or 25 °C using an MOI of 100. The efficiency of the phage cocktail was found to be relatively lower at 4°C, when compared with that at 25 °C. Storage at a lower temperature (4 °C) could prevent the regrowth of the host bacteria after phage treatment [41,89]. Previous studies detected a significant reduction in the recovered *S.* Enteritidis by up to 3 log_10_ while using phage cocktail at an MOI of 10000 and 1000 in milk, chicken breast, and cabbage detected [62,83], and the application of phage cocktails has also been shown to reduce *Salmonella* spp. in different food matrices [26,39,90,91,92,93,94,95].

The biofilm assay has shown that the phage cocktail has the potential to eradicate and reduce the biofilm formed by S. Typhimurium EG.SmT3. The results indicated that the phage cocktail eradicated post-treated biofilm in 96-well microplate (64.34–74.26%) at the tested titers. A previous study on *S*. Typhimurium and *S*. Enteritidis showed significant biofilm eradications in the 96-well microplate (44–63%) [62]. The results in this study demonstrated the efficacy of lytic *Salmonella* phage cocktail to combat the multi-drug resistant S. Typhimurium in milk, water, and chicken breast. The established features of isolated phages in this study have shown that they could potentially reduce Salmonellosis in ready-to-eat food and reduce biofilms on food contact surfaces that are very crucial in maintaining public health.

## Figures and Tables

**Table 1:** The efficiency of plating (EOP) by phages SPHG1, SPHG3, and cocktail mix of these phages against different bacteria.

Species	Strain ID Number	Lysis by Bacteriophage		
		Phage SPHG1	Phage SPHG3	Phage Cocktail
***S*** **. Typhimurium**	EG.SmT1	0.67	0.88	0.92
	EG.SmT2	1	0.93	1
	EG.SmT3 (phages enrichment host)	Host	Host	Host
	101SM	0.002	0.27	0.89
***S*** **. Enteritidis**	EG.SmE1	0	0.02	0.20
	EG.SmE2	0	0.2	0.25
	EG.SE1	0	0	0
	331SM	0.04	0.18	0.5
***S*** **. Kentucky**	7	0.12	0.004	0.42
	12	0.013	0.005	0.24
	51	0	0.10	0.44
***S*** **. Typhi**	SamTph1	0	0.16	0.34
	SamTph2	0	0.21	0.27
	SamTph5	0	0.09	0.12
***S. para*** **Typhi**	102	0	0	0
***E. coli***	BE1	0	0	0
	BE2	0	0	0
	BE3	0	0	0
***S. aureus***	SA101	0	0	0
	SA1E	0	0	0
	EG-AE1	0	0	0

High efficiency, EOP 0.5 to 1.0; moderate efficiency, EOP 0.2 to <0.5; low efficiency, 0.0001 to <0.2; and inefficient <0.001.

## Data Availability

No applicable.

## Supplementary Materials

The following are available online at https://www.mdpi.com/2076-2607/9/2/423/s1, Table S1: Bacterial strains used in current study, Table S2: BLASTn alignment of homologs phage sequences (at the GenBank database) with SPHG1, Table S3: Genome annotation of the SPHG1 genome, Table S4: SBLASTn alignment of homologs phage sequences (at the GenBank database) with phage SPHG3, Table S5: Genome annotation of the SPHG3 genome.

## References

[1] WHO World Health Organization (2017). https://www.who.int/foodsafety/areas_work/foodborne-diseases/salmonella/en/.

[2] Musyoka J.N., Abong G.O., Mbogo D.M., Fuchs R., Low J., Heck S., Muzhingi T. (2018). Effects of Acidification and Preservatives on Microbial Growth during Storage of Orange Fleshed Sweet Potato Puree. Int. J. Food Sci..

[3] Majowicz S.E., Musto J., Scallan E., Angulo F.J., Kirk M., O’Brien S.J., Jones T.F., Fazil A., Hoekstra R.M. (2010). The Global Burden of Nontyphoidal Salmonella Gastroenteritis. Clin. Infect. Dis..

[4] Hanning I.B., Nutt J.D., Ricke S.C. (2009). Salmonellosis outbreaks in the united states due to fresh produce: Sources and potential intervention measures. Foodborne Pathog. Dis..

[5] (2016). CDC Salmonella Homepage. https://www.cdc.gov/salmonella/index.html.

[6] Humphrey T. (2004). *Salmonella*, stress responses and food safety. Nat. Rev. Microbiol..

[7] Spector M.P., Kenyon W.J. (2012). Resistance and survival strategies of *Salmonella* enterica to environmental stresses. Food Res. Int..

[8] Lamas A., Regal P., Vázquez B., Miranda J.M., Cepeda A., Franco C.M. (2018). *Salmonella* and Campylobacter biofilm formation: A comparative assessment from farm to fork. J. Sci. Food Agric..

[9] Corcoran M., Morris D., De Lappe N., O’Connor J., Lalor P., Dockery P., Cormican M. (2014). Commonly used disinfectants fail to eradicate *Salmonella* enterica biofilms from food contact surface materials. Appl. Environ. Microbiol..

[10] Khatoon Z., McTiernan C.D., Suuronen E.J., Mah T.F., Alarcon E.I. (2018). Bacterial biofilm formation on implantable devices and approaches to its treatment and prevention. Heliyon.

[11] Chylkova T., Cadena M., Ferreiro A., Pitesky M. (2017). Susceptibility of *Salmonella* biofilm and planktonic bacteria to common disinfectant agents used in poultry processing. J. Food Prot..

[12] Neetoo H., Mahomoodally F. (2014). Use of antimicrobial films and edible coatings incorporating chemical and biological preservatives to control growth of Listeria monocytogenes on cold smoked salmon. BioMed Res. Int..

[13] Pérez-Díaz I.M., Truong V.-D., Webber A., McFeeters R.F. (2008). Microbial growth and the effects of mild acidification and preservatives in refrigerated sweet potato puree. J. Food Prot..

[14] Latou E., Mexis S.F., Badeka A.V., Kontominas M.G. (2010). Shelf life extension of sliced wheat bread using either an ethanol emitter or an ethanol emitter combined with an oxygen absorber as alternatives to chemical preservatives. J. Cereal Sci..

[15] Khoshnoud M.J., Siavashpour A., Bakhshizadeh M., Rashedinia M. (2018). Effects of sodium benzoate, a commonly used food preservative, on learning, memory, and oxidative stress in brain of mice. J. Biochem. Mol. Toxicol..

[16] Pawlowska A.M., Zannini E., Coffey A., Arendt E.K. (2012). “Green Preservatives”: Combating Fungi in the Food and Feed Industry by Applying Antifungal Lactic Acid Bacteria. Advances in Food and Nutrition Research.

[17] Asadullah, Khair-un-Nisa, Tarar O.M., Ali S.A., Jamil K., Begum A. (2010). Study to evaluate the impact of heat treatment on water soluble vitamins in milk. J. Pak. Med. Assoc..

[18] Lešková E., Kubíková J., Kováčiková E., Košická M., Porubská J., Holčíková K. (2006). Vitamin losses: Retention during heat treatment and continual changes expressed by mathematical models. J. Food Compos. Anal..

[19] Erbersdobler H.F., Faist V. (2001). Metabolic transit of Amadori products. Nahr. Food.

[20] Agyare C., Etsiapa Boamah V., Ngofi Zumbi C., Boateng Osei F. (2019). Antibiotic Use in Poultry Production and Its Effects on Bacterial Resistance. Antimicrobial Resistance—A Global Threat.

[21] Medeiros M.A.N., De Oliveira D.C.N., Dos Prazeres Rodrigues D., De Freitas D.R.C. (2011). Prevalence and antimicrobial resistance of *Salmonella* in chicken carcasses at retail in 15 Brazilian cities. Rev. Panam. Salud Publica.

[22] Casewell M., Friis C., Marco E., McMullin P., Phillips I. (2003). The European ban on growth-promoting antibiotics and emerging consequences for human and animal health. J. Antimicrob. Chemother..

[23] Dijksterhuis J., Samson R.A. (2006). Zygomycetes. Food Spoilage Microorganisms.

[24] Matsuzaki S., Uchiyama J., Takemura-Uchiyama I., Daibata M. (2014). Perspective: The age of the phage. Nature.

[25] Lin D.M., Koskella B., Lin H.C. (2017). Phage therapy: An alternative to antibiotics in the age of multi-drug resistance. World J. Gastrointest. Pharmacol. Ther..

[26] Goodridge L.D., Bisha B. (2011). Phage-based biocontrol strategies to reduce foodborne pathogens in foods. Bacteriophage.

[27] Moye Z.D., Woolston J., Sulakvelidze A. (2018). Bacteriophage applications for food production and processing. Viruses.

[28] Doss J., Culbertson K., Hahn D., Camacho J., Barekzi N. (2017). A review of phage therapy against bacterial pathogens of aquatic and terrestrial organisms. Viruses.

[29] Salmond G.P.C., Fineran P.C. (2015). A century of the phage: Past, present and future. Nat. Rev. Microbiol..

[30] Rohwer F., Segall A.M. (2015). In retrospect: A century of phage lessons. Nature.

[31] Tan L.-H., Chan K.-G., Lee L.-H. (2014). Application of Bacteriophage in Biocontrol of Major Foodborne Bacterial Pathogens. J. Mol. Biol. Mol. Imaging.

[32] Sulakvelidze A. (2013). Using lytic bacteriophages to eliminate or significantly reduce contamination of food by foodborne bacterial pathogens. J. Sci. Food Agric..

[33] Paul H., Abedon S.T. (2012). Bacteriophages in Health and Disease.

[34] De Paepe M., Leclerc M., Tinsley C.R., Petit M.A. (2014). Bacteriophages: An underestimated role in human and animal health?. Front. Cell. Infect. Microbiol..

[35] Tiwari R., Dhama K., Chakraborty S., Kumar A., Rahal A., Kapoor S. (2014). Bacteriophage therapy for safeguarding animal and human health: A review. Pak. J. Biol. Sci..

[36] Kutter E., De Vos D., Gvasalia G., Alavidze Z., Gogokhia L., Kuhl S., Abedon S. (2010). Phage Therapy in Clinical Practice: Treatment of Human Infections. Curr. Pharm. Biotechnol..

[37] Keen E.C., Adhya S.L., Wormser G.P. (2015). Phage therapy: Current research and applications. Clin. Infect. Dis..

[38] Augustine J., Bhat S.G. (2015). Biocontrol of *Salmonella* Enteritidis in spiked chicken cuts by lytic bacteriophages ΦSP-1 and ΦSP-3. J. Basic Microbiol..

[39] Spricigo D.A., Bardina C., Cortés P., Llagostera M. (2013). Use of a bacteriophage cocktail to control *Salmonella* in food and the food industry. Int. J. Food Microbiol..

[40] Hungaro H.M., Mendonça R.C.S., Gouvêa D.M., Vanetti M.C.D., Pinto C.L.O. (2013). Use of bacteriophages to reduce *Salmonella* in chicken skin in comparison with chemical agents. Food Res. Int..

[41] Bigwood T., Hudson J.A., Billington C., Carey-Smith G.V., Heinemann J.A. (2008). Phage inactivation of foodborne pathogens on cooked and raw meat. Food Microbiol..

[42] Hooton S.P.T., Atterbury R.J., Connerton I.F. (2011). Application of a bacteriophage cocktail to reduce *Salmonella* Typhimurium U288 contamination on pig skin. Int. J. Food Microbiol..

[43] Kocharunchitt C., Ross T., McNeil D.L. (2009). Use of bacteriophages as biocontrol agents to control *Salmonella* associated with seed sprouts. Int. J. Food Microbiol..

[44] Modi R., Hirvi Y., Hill A., Griffiths M.W. (2001). Effect of phage on survival of *Salmonella* Enteritidis during manufacture and storage of Cheddar cheese made from raw and pasteurized milk. J. Food Prot..

[45] Phothaworn P., Supokaivanich R., Lim J., Klumpp J., Imam M., Kutter E., Galyov E.E., Dunne M., Korbsrisate S. (2020). Development of a broad-spectrum *Salmonella* phage cocktail containing Viunalike and Jerseylike viruses isolated from Thailand. Food Microbiol..

[46] Endersen L., O’Mahony J., Hill C., Ross R.P., McAuliffe O., Coffey A. (2014). Phage therapy in the food industry. Annu. Rev. Food Sci. Technol..

[47] Hagens S., Loessner M. (2010). Bacteriophage for Biocontrol of Foodborne Pathogens: Calculations and Considerations. Curr. Pharm. Biotechnol..

[48] Clokie M.R.J., Kropinski A.M. (2009). Bacteriophages: Methods and Protocols.

[49] Huang C., Shi J., Ma W., Li Z., Wang J., Li J., Wang X. (2018). Isolation, characterization, and application of a novel specific *Salmonella* bacteriophage in different food matrices. Food Res. Int..

[50] Jun J.W., Kim J.H., Shin S.P., Han J.E., Chai J.Y., Park S.C. (2013). Protective effects of the Aeromonas phages pAh1-C and pAh6-C against mass mortality of the cyprinid loach (Misgurnus anguillicaudatus) caused by Aeromonas hydrophila. Aquaculture.

[51] Akhtar M., Viazis S., Diez-Gonzalez F. (2014). Isolation, identification and characterization of lytic, wide host range bacteriophages from waste effluents against *Salmonella* enterica serovars. Food Control.

[52] Van Twest R., Kropinski A.M. (2009). Bacteriophage enrichment from water and soil. Methods Mol. Biol..

[53] Kropinski A.M., Mazzocco A., Waddell T.E., Lingohr E., Johnson R.P. (2009). Enumeration of bacteriophages by double agar overlay plaque assay. Methods Mol. Biol..

[54] Melo L.D.R., Ferreira R., Costa A.R., Oliveira H., Azeredo J. (2019). Efficacy and safety assessment of two enterococci phages in an in vitro biofilm wound model. Sci. Rep..

[55] Melo L.D.R., Veiga P., Cerca N., Kropinski A.M., Almeida C., Azeredo J., Sillankorva S. (2016). Development of a phage cocktail to control Proteus mirabilis catheter-associated urinary tract infections. Front. Microbiol..

[56] Huang C., Virk S.M., Shi J., Zhou Y., Willias S.P., Morsy M.K., Abdelnabby H.E., Liu J., Wang X., Li J. (2018). Isolation, characterization, and application of Bacteriophage LPSE1 against *Salmonella* enterica in Ready to Eat (RTE) Foods. Front. Microbiol..

[57] Mirzaei M.K., Nilsson A.S. (2015). Isolation of phages for phage therapy: A comparison of spot tests and efficiency of plating analyses for determination of host range and efficacy. PLoS ONE.

[58] Ackermann H.W. (2012). Bacteriophage Electron Microscopy. Advances in Virus Research.

[59] Islam M.S., Raz A., Liu Y., Elbassiony K.R.A., Dong X., Zhou P., Zhou Y., Li J. (2019). Complete Genome Sequence of Aeromonas Phage ZPAH7 with Halo Zones, Isolated in China. Microbiol. Resour. Announc..

[60] Lowe T.M., Eddy S.R. (1997). tRNAscan-SE: A program for improved detection of transfer RNA genes in genomic sequence. Nucleic Acids Res..

[61] Stothard P., Wishart D.S. (2005). Circular genome visualization and exploration using CGView. Bioinformatics.

[62] Islam M.S., Zhou Y., Liang L., Nime I., Liu K., Yan T., Wang X., Li J. (2019). Application of a Phage Cocktail for Control of *Salmonella* in Foods and Reducing Biofilms. Viruses.

[63] Kostaki M., Chorianopoulos N., Braxou E., Nychas G.J., Giaouris E. (2012). Differential biofilm formation and chemical disinfection resistance of sessile cells of Listeria monocytogenes strains under monospecies and dual-species (with *Salmonella* enterica) conditions. Appl. Environ. Microbiol..

[64] Eurosurveillance editorial team (2012). The European Union summary report on trends and sources of zoonoses, zoonotic agents and food-borne outbreaks in 2010. Eurosurveillance.

[65] Abdelhakim A., Yamina M., Mohammed S.S., Hala M.A., Mervat G.E.A., Souhila A., Rabah B. (2011). Resistance to -lactams of human and veterinary *Salmonella* isolates in Egypt and Algeria. Afr. J. Microbiol. Res..

[66] Wasfy M.O., Frenck R., Ismail T.F., Mansour H., Malone J.L., Mahoney F.J. (2002). Trends of Multiple-Drug Resistance among *Salmonella* Serotype Typhi Isolates during a 14-Year Period in Egypt. Clin. Infect. Dis..

[67] Abdel-Maksoud M., Abdel-Khalek R., El-Gendy A., Gamal R.F., Abdelhady H.M., House B.L. (2015). Genetic characterisation of multidrug-resistant *Salmonella* enterica serotypes isolated from poultry in Cairo, Egypt. Afr. J. Lab. Med..

[68] Merwad A.M.A., Abdel-Haliem M.E.F. (2018). ISOLATION AND INITIAL CHARACTERIZATION OF A Myoviridae PHAGE FOR CONTROLLING ZOONOTIC *Salmonella* Typhimurium AND *Salmonella* Enteritidis FROM BROILERS IN EGYPT. Slov. Vet. Res..

[69] Mahmoud M., Askora A., Barakat A.B., Rabie O.E.F., Hassan S.E. (2018). Isolation and characterization of polyvalent bacteriophages infecting multi drug resistant *Salmonella* serovars isolated from broilers in Egypt. Int. J. Food Microbiol..

[70] Pires D.P., Oliveira H., Melo L.D.R., Sillankorva S., Azeredo J. (2016). Bacteriophage-encoded depolymerases: Their diversity and biotechnological applications. Appl. Microbiol. Biotechnol..

[71] Petty N.K., Evans T.J., Fineran P.C., Salmond G.P.C. (2007). Biotechnological exploitation of bacteriophage research. Trends Biotechnol..

[72] Bielke L., Higgins S., Donoghue A., Donoghue D., Hargis B.M. (2007). *Salmonella* host range of bacteriophages that infect multiple genera. Poult. Sci..

[73] O’Flynn G., Coffey A., Fitzgerald G.F., Ross R.P. (2006). The newly isolated lytic bacteriophages st104a and st104b are highly virulent against *Salmonella* enterica. J. Appl. Microbiol..

[74] Tanji Y., Shimada T., Yoichi M., Miyanaga K., Hori K., Unno H. (2004). Toward rational control of Escherichia coli O157:H7 by a phage cocktail. Appl. Microbiol. Biotechnol..

[75] Bai J., Jeon B., Ryu S. (2019). Effective inhibition of *Salmonella* Typhimurium in fresh produce by a phage cocktail targeting multiple host receptors. Food Microbiol..

[76] Chan B.K., Abedon S.T., Loc-Carrillo C. (2013). Phage cocktails and the future of phage therapy. Future Microbiol..

[77] Mahony J., McAuliffe O., Ross R.P., van Sinderen D. (2011). Bacteriophages as biocontrol agents of food pathogens. Curr. Opin. Biotechnol..

[78] Omwandho C.O.A., Kubota T. (2010). *Salmonella* enterica serovar Enteritidis: A mini-review of contamination routes and limitations to effective control. Jpn. Agric. Res. Q..

[79] Nováček J., Šiborová M., Benešík M., Pantůček R., Doškař J., Plevka P. (2016). Structure and genome release of Twort-like Myoviridae phage with a double-layered baseplate. Proc. Natl. Acad. Sci. USA.

[80] Sunderland K.S., Yang M., Mao C. (2017). Phage-Enabled Nanomedicine: From Probes to Therapeutics in Precision Medicine. Angew. Chem. Int. Ed..

[81] Guenther S., Herzig O., Fieseler L., Klumpp J., Loessner M.J. (2012). Biocontrol of *Salmonella* Typhimurium in RTE foods with the virulent bacteriophage FO1-Eint. J. Food Microbiol..

[82] Chan B.K., Abedon S.T. (2012). Phage therapy pharmacology. Phage cocktails. Advances in Applied Microbiology.

[83] Bao H., Zhang P., Zhang H., Zhou Y., Zhang L., Wang R. (2015). Bio-control of *salmonella* enteritidis in foods using bacteriophages. Viruses.

[84] Andreatti Filho R.L., Higgins J.P., Higgins S.E., Gaona G., Wolfenden A.D., Tellez G., Hargis B.M. (2007). Ability of bacteriophages isolated from different sources to reduce *Salmonella* enterica serovar Enteritidis in vitro and in vivo. Poult. Sci..

[85] Duc H.M., Son H.M., Honjoh K.-I., Miyamoto T. (2018). Isolation and application of bacteriophages to reduce *Salmonella* contamination in raw chicken meat. Lwt Food Sci. Technol..

[86] El-Shibiny A., El-Sahhar S., Adel M. (2017). Phage applications for improving food safety and infection control in Egypt. J. Appl. Microbiol..

[87] Abedon S. (2011). Phage Therapy Pharmacology. Calculating Phage Dosing. Advances in Applied Microbiology.

[88] Abedon S.T. (2011). Lysis from without. Bacteriophage.

[89] Guenther S., Loessner M.J. (2011). Bacteriophage biocontrol of Listeria monocytogenes on soft ripened white mold and red-smear cheeses. Bacteriophage.

[90] Sharma M., Dashiell G., Handy E.T., East C., Reynnells R., White C., Nyarko E., Micallef S., Hashem F., Millner P.D. (2017). Survival of *Salmonella* newport on whole and fresh-cut cucumbers treated with lytic bacteriophages. J. Food Prot..

[91] Grant A., Hashem F., Parveen S. (2016). *Salmonella* and Campylobacter: Antimicrobial resistance and bacteriophage control in poultry. Food Microbiol..

[92] Kang H.W., Kim J.W., Jung T.S., Woo G.J. (2013). wksl3, a new biocontrol agent for *Salmonella* enterica serovars enteritidis and typhimurium in foods: Characterization, application, sequence analysis, and oral acute toxicity study. Appl. Environ. Microbiol..

[93] Heyse S., Hanna L.F., Woolston J., Sulakvelidze A., Charbonneau D. (2015). Bacteriophage cocktail for biocontrol of *Salmonella* in dried pet food. J. Food Prot..

[94] Ye J., Kostrzynska M., Dunfield K., Warriner K. (2009). Evaluation of a Biocontrol Preparation Consisting of Enterobacter asburiae JX1 and a Lytic Bacteriophage Cocktail To Suppress the Growth of *Salmonella* Javiana Associated with Tomatoes. J. Food Prot..

[95] López-Cuevas O., Campo N.C., Ramirez K., Chaidez C. (2016). Biocontrol of *Salmonella* Typhimurium growth in tomato surface by bacteriophage P22. Afr. J. Microbiol. Res..

