# Supplementary material for: Isolation and Characterization of Two Lytic Bacteriophages Infecting a Multi-Drug Resistant Salmonella Typhimurium and Their Efficacy to Combat Salmonellosis in Ready-to-Use Foods
Source: Microorganisms. 2021 Feb 18;9(2):423. doi: 10.3390/microorganisms9020423 (PMC7922427; doi:10.3390/microorganisms9020423)
Supplement: Supplementary file 1 [file microorganisms-09-00423-s001.pdf]

microorganisms

Supplementary Materials

Isolation and characterization of two lytic bacteriophages infecting a multi-drug resistant *Salmonella* Typhimurium and their efficacy to combat salmonellosis in ready-to-use foods

Ahmed Esmael [1,*], Ehab Azab [2], Adil A. Gobouri [3], Mohamed A. Nasr-Eldin [1], Mahmoud M.A. Moustafa [4], Shereen A. Mohamed [4], Omnia A. M. Badr [4] and Alzahraa M. Abdelatty [5,*]

[1] Botany and Microbiology Department, Faculty of Science, Benha University, Qalubiya Governorate, 13511, Egypt; ahmed.esmael@fsc.bu.edu.eg (A.E); nasreldeen.m@gmail.com (M.A.N.)
[2] Department of Nutrition and Food Science, College of Science, Taif University, P.O. Box 11099, Taif 21944, Saudia Arabia; e.azab@tu.edu.sa (E.A.)
[3] Department of Chemistry, College of Science, Taif University, P.O. Box 11099, Taif 21944, Saudia Arabia; a.gobouri@tu.edu.sa (A.A.G.)
[4] Department of Genetics and Genetic Engineering, Faculty of Agriculture, Benha University, Qalubiya Governorate, 13736, Egypt; mahmoud.mustafa@fagr.bu.edu.eg (M.M.A.M); shereen.mustafa@fagr.bu.edu.eg (S.A.M); omnia.badr@fagr.bu.edu.eg (O.A.M.B)
[5] Department of Nutrition and Clinical Nutrition, Faculty of Veterinary Medicine, Cairo University, Giza, Egypt; alzahraa@cu.edu.eg; raramehmet@gmail.com (A.M.A)
* Correspondence: ahmed.esmael@fsc.bu.edu.eg (A.E.), alzahraa@cu.edu.eg ; raramehmet@gmail.com (A.M.A.)

Table S1. Bacterial strains used in current study.

Species	Strain ID number	Source
***S.* Typhimurium**	EG.SmT1	AE[1]
	EG.SmT2	AE
	EG.SmT3 (phages enrichment host)	AE
	101SM	FSCBU[2]
***S.* Enteritidis**	EG.SmE1	A.E.
	EG.SmE2	A.E.
	EG.SE1	FSCBU
	331SM	FSCBU
***S.* Kentucky**	7	FSCBU
	12	FSCBU
	51	FSCBU
***S.* Typhi**	SamTph1	FSCBU
	SamTph2	FSCBU
	SamTph5	FSCBU
***S.* para Typhi**	102	FSCBU
E. coli	BE1	FSCBU
	BE2	FSCBU
	BE3	FSCBU
***S.* aureus**	SA101	FSCBU
	SA1E	FSCBU
	EG-AE1	FSCBU
Number (N=21)		

[1] AE (From poultry farm with a history of diarrhea).

[2] FSCBU (Microbiology lab, Botany and Microbiology Dept., Faculty of Science, Benha Univ.).

Table S2. BLASTn alignment of homologs phage sequences (at the GenBank database) with Salmonella phage SPHG1

Phage	Phage Family	Isolated place	Accession#	Length (bp)	E value	Query coverage
**Salmonella phage vB_SenM-1**	*Myoviridae*	Poland	MT012730.1	52471	0.0	97%
**Salmonella phage SE13**	*Myoviridae*	Canada	NC_048763.1	52438	0.0	94%
**Salmonella phage brorfarstad**	*Myoviridae*	Denmark	MT074435.1	52392	0.0	95%
**Salmonella phage LSE7621**	*Myoviridae*	China	MK568062.1	50936	0.0	88%
**Salmonella phage BP63**	*Myoviridae*	USA	KM366099.1	52437	0.0	90%

Table S3. Genome annotation of SPHG1 genome

CDS	Gene Position	Direction	Putative Function
SPHG1_01	1- 861	Fwr.	Thymidylate synthase
SPHG1_02	894- 1139	Rev.	Hypothetical protein
SPHG1_03	1170- 1589	Rev.	Hypothetical protein
SPHG1_04	1620- 4112	Rev.	DNA primase/ helicase
SPHG1_05	4128- 4772	Fwr.	Hypothetical protein
SPHG1_06	5043- 6665	Rev.	superfamily II helicase
SPHG1_07	6742- 7584	Rev.	ATP-binding protein
SPHG1_08	7601- 7849	Rev.	Hypothetical protein
SPHG1_09	7973- 8389	Rev.	Hypothetical protein
SPHG1_10	8438- 9340	Rev.	Hypothetical protein
SPHG1_11	9455- 9745	Rev.	Hypothetical protein
SPHG1_12	9790- 10,338	Fwr.	Hypothetical protein
SPHG1_13	10,521- 11,018	Fwr.	Hypothetical protein
SPHG1_14	11,015- 11,356	Fwr.	Hypothetical protein
SPHG1_15	11,429- 11,878	Fwr.	Hypothetical protein
SPHG1_16	11,923- 12,234	Fwr.	Hypothetical protein
SPHG1_17	12,467- 12,748	Rev.	Hypothetical protein
SPHG1_18	12,770- 13,165	Rev.	Hypothetical protein
SPHG1_19	13,232- 13,351	Rev.	Hypothetical protein
SPHG1_20	13,376- 13,570	Rev.	Hypothetical protein
SPHG1_21	13,598- 13,723	Rev.	Hypothetical protein
SPHG1_22	13,760- 13,885	Fwr.	Hypothetical protein
SPHG1_23	13,907- 14,323	Rev.	Hypothetical protein
SPHG1_24	14,392- 14,613	Rev.	Hypothetical protein
SPHG1_25	15,099- 15,395	Rev.	Hypothetical protein
SPHG1_26	15,491- 15,646	Rev.	Hypothetical protein
SPHG1_27	16,220- 16,357	Fwr.	Hypothetical protein
SPHG1_28	16,342- 16,461	Fwr.	Hypothetical protein

SPHG1_29	16,504- 16,986	Fwr.	Hypothetical protein
SPHG1_30	17,028- 17,690	Fwr.	dihydrofolate reductase
SPHG1_31	17,687- 18,217	Fwr.	guanylate kinase
SPHG1_32	18,214- 18,960	Fwr.	Hypothetical protein
SPHG1_33	18,960- 19,274	Fwr.	Hypothetical protein
SPHG1_34	19,372- 20,865	Fwr.	terminase large subunit
SPHG1_35	20,888- 21,178	Rev.	Hypothetical protein
SPHG1_36	21,240- 22,832	Fwr.	portal protein
SPHG1_37	22,850- 23,635	Rev.	Major capsid protein
SPHG1_38	23,678- 24,382	Rev.	Scaffold protein
SPHG1_39	24,413- 24,895	Rev.	Hypothetical protein
SPHG1_40	24,819- 25,196	Rev.	Hypothetical protein
SPHG1_41	25,196- 25,684	Rev.	Hypothetical protein
SPHG1_42	25,684- 26,124	Rev.	Hypothetical protein
SPHG1_43	26,049- 26,987	Rev.	Major capsid protein
SPHG1_44	27,129- 27,803	Fwr.	Putative tail fibers protein
SPHG1_45	27,804- 30,767	Fwr.	Tail formation protein
SPHG1_46	30,768- 31,115	Rev.	Hypothetical protein
SPHG1_47	31,127- 33,031	Fwr.	major tail protein
SPHG1_48	33,124- 33,909	Fwr.	Hypothetical protein
SPHG1_49	33,978- 35,657	Fwr.	Tail tape measure protein
SPHG1_50	35,681- 36,541	Fwr.	Hypothetical protein
SPHG1_51	36,562- 37,209	Fwr.	Translation initiation factor IF-2
SPHG1_52	37,219- 38,178	Fwr.	Hypothetical protein
SPHG1_53	38,179- 38,523	Fwr.	Hypothetical protein
SPHG1_54	38,523- 39,674	Fwr.	Hypothetical protein
SPHG1_55	39,675- 41,060	Fwr.	tail fiber protein
SPHG1_56	41,077- 41,652	Fwr.	Putative tail fibers protein
SPHG1_57	41,662- 42,144	Fwr.	Putative tail fibers protein
SPHG1_58	42,224- 42,847	Fwr.	Cof hydrolase
SPHG1_59	42,875- 43,141	Rev.	Hypothetical protein
SPHG1_60	43,162- 43,668	Fwr.	Lysin
SPHG1_61	43,669- 44,628	Rev.	Hypothetical protein
SPHG1_62	44,658- 46,616	Fwr.	DNA polymerase I

Table S4. BLASTn alignment of homologs phage sequences (at the GenBank database) with SPHG3

Phage	Phage Family	Isolated place	Accession#	Length (bp)	E value	Query coverage
**Salmonella phage ST-W77**	*Ackermannviridae*	Thailand	NC_049378.1	157458	0.0	99%
**Salmonella phage Se_AO1**	*Ackermannviridae*	Spain	LR800396.1	157543	0.0	91%
**Salmonella phage vB_SalM_PM10**	*Ackermannviridae*	India	NC_031128.1	158081	0.0	91%
**Salmonella phage chennai**	*Ackermannviridae*	India	MN953776.1	157462	0.0	91%
**Salmonella phage kage**	*Ackermannviridae*	Denmark	MT074476.1	157658	0.0	92%

Table S5. Genome annotation of the SPHG3 genome

CDS	Gene Position	Direction	Putative Function
SPHG3_01	14-763	Rev.	Hypothetical protein
SPHG3_02	763-1221	Rev.	Putative DNA repair/recombination protein
SPHG3_03	1260-1757	Rev.	Tail completion protein
SPHG3_04	2442-3173	Rev.	Hypothetical protein
SPHG3_05	3410-3832	Rev.	Hypothetical protein
SPHG3_06	4224-4688	Rev.	Hypothetical protein
SPHG3_07	4792-5046	Rev.	Hypothetical protein
SPHG3_08	5779-6228	Rev.	Hypothetical protein
SPHG3_09	6437-6673	Rev.	Hypothetical protein
SPHG3_10	6773-8095	Rev.	Major head protein
SPHG3_11	8187-9026	Rev.	Prohead assembly (scaffolding) protein
SPHG3_12	9073-9741	Rev.	prohead core and protease
SPHG3_13	9749-10,066	Rev.	Hypothetical protein
SPHG3_14	10,287-11,969	Rev.	Portal vertex protein of head
SPHG3_15	12,037-12,570	Rev.	Phage tail tube monomer
SPHG3_16	12,601-13,059	Rev.	Hypothetical protein
SPHG3_17	13,118-13,438	Rev.	Tail sheath protein
SPHG3_18	13,486-15,012	Rev.	Tail sheath protein
SPHG3_19	15,065-17,275	Rev.	Terminase DNA packaging enzyme large subunit
SPHG3_20	17,504-17,935	Rev.	Gp16 terminase DNA packaging enzyme small subunit
SPHG3_21	17,938-18,636	Rev.	Proximal tail sheath stabilization protein
SPHG3_22	18,639-19,280	Rev.	Phage neck protein
SPHG3_23	19,583-20,392	Rev.	Phage Neck protein
SPHG3_24	20,322-20,663	Rev.	Hypothetical protein
SPHG3_25	20,647-20,943	Rev.	Hypothetical protein
SPHG3_26	21,052-22,446	Rev.	Hypothetical protein
SPHG3_27	22,374-23,585	Rev.	Tail fibers protein
SPHG3_28	23,637-24,329	Rev.	Hypothetical protein
SPHG3_29	24,326-26,416	Rev.	Hypothetical protein
SPHG3_30	26,514-27,956	Rev.	Phage tail fibers
SPHG3_31	28,433-28,639	Rev.	Phage tail fibers
SPHG3_32	28,719-31,760	Rev.	Phage tail fibers
SPHG3_33	31,807-32,997	Rev.	Hypothetical protein

SPHG3_34	33,000-33,881	Rev.	Hypothetical protein
SPHG3_35	33,838-35,631	Rev.	Baseplate wedge subunit
SPHG3_36	37,092-37,352	Rev.	Hypothetical protein
SPHG3_37	37,887-38,135	Rev.	Hypothetical protein
SPHG3_38	38,893-38,965	Fwr.	tRNA-Met
SPHG3_39	39,381-39,453	Fwr.	tRNA-Asn
SPHG3_40	40,213-40,296	Fwr.	tRNA-Tyr
SPHG3_41	40,376-40,461	Fwr.	tRNA-Ser
SPHG3_42	40,468-40,557	Fwr.	tRNA-other
SPHG3_43	40,558-40,782	Rev.	Hypothetical protein
SPHG3_44	40,927-41,226	Fwr.	Hypothetical protein
SPHG3_45	44,172-44,546	Rev.	Hypothetical protein
SPHG3_46	44,543-45,100	Rev.	Hypothetical protein
SPHG3_47	45,297-46,034	Rev.	Hypothetical protein
SPHG3_48	46,204-46,794	Rev.	Hypothetical protein
SPHG3_49	46,856-49,852	Fwr.	DNA polymerase
SPHG3_50	49,853-50,917	Rev.	DNA Primase
SPHG3_51	50,914-50,917	Rev.	Hypothetical protein
SPHG3_52	50,914-51,372	Rev.	N-acetylmuramoyl-L-alanine amidase (Autolysin)
SPHG3_53	51,685-52,188	Rev.	Hypothetical protein
SPHG3_54	52,557-52,850	Rev.	Hypothetical protein
SPHG3_55	52,861-53,478	Rev.	Hypothetical protein
SPHG3_56	53,481-53,846	Rev.	Hypothetical protein
SPHG3_57	53,850-56,183	Rev.	Recombination-related endonuclease
SPHG3_58	56,186-57,301	Rev.	Recombination-related endonuclease
SPHG3_59	57,288-58,079	Rev.	Sigma factor involved in late transcription
SPHG3_60	58,091-58,618	Rev.	Ribonucleasse H
SPHG3_61	59,425-61,089	Rev.	Probable ATP-dependent helicase
SPHG3_62	61,282-61,560	Rev.	DNA-binding protein
SPHG3_63	61,644-62,435	Rev.	Hypothetical protein
SPHG3_64	62,641-62,961	Rev.	Superinfection exclusion protein
SPHG3_65	62,951-63,238	Rev.	Hypothetical protein
SPHG3_66	63,240-63,908	Rev.	Hypothetical protein
SPHG3_67	63,965-64,756	Rev.	Hypothetical protein
SPHG3_68	64,806-65,126	Rev.	Hypothetical protein
SPHG3_69	65,123-65,812	Rev.	Hypothetical protein
SPHG3_70	66,379-66,657	Rev.	Hypothetical protein
SPHG3_71	66,670-67,173	Rev.	Endonuclease
SPHG3_72	67,185-67,424	Rev.	Hypothetical protein
SPHG3_73	67,556-67,873	Rev.	Hypothetical protein
SPHG3_74	67,911-70,127	Rev.	Hypothetical protein
SPHG3_75	70,292-70,651	Rev.	Hypothetical protein
SPHG3_76	70,751-71,476	Rev.	Hypothetical protein
SPHG3_77	71,469-71,720	Rev.	Hypothetical protein
SPHG3_78	72,102-72,338	Rev.	Hypothetical protein
SPHG3_79	72,398-72,769	Rev.	Hypothetical protein

SPHG3_80	72,808-74,823	Rev.	Hypothetical protein
SPHG3_81	74,939-76,051	Rev.	Hypothetical protein
SPHG3_82	76,228-77,181	Rev.	Hypothetical protein
SPHG3_83	77,111-77,575	Rev.	Translation repressor protein
SPHG3_84	77,605-78,027	Rev.	DNA polymerase clamp loader subunit
SPHG3_85	78,032-79,021	Rev.	DNA polymerase clamp loader subunit
SPHG3_86	79,100-79,768	Rev.	Sliding clamp DNA polymerase accessory protein
SPHG3_87	80,479-81,978	Rev.	DNA helicase
SPHG3_88	82,039-82,383	Fwr.	Hypothetical protein
SPHG3_89	82,376-83,158	Fwr.	Hypothetical protein
SPHG3_90	83,474-84,379	Fwr.	Hypothetical protein
SPHG3_91	84,921-86,162	Fwr.	Hypothetical protein
SPHG3_92	86,311-91,154	Fwr.	similar to virulence-associated VriC protein
SPHG3_93	92,095-93,204	Fwr.	Hypothetical protein
SPHG3_94	93,220-93,789	Fwr.	Hypothetical protein
SPHG3_95	93,794-94,216	Fwr.	Hypothetical protein
SPHG3_96	94,266-94,649	Fwr.	Hypothetical protein
SPHG3_97	94,646-95,092	Fwr.	Hypothetical protein
SPHG3_98	95,064-95,291	Fwr.	Hypothetical protein
SPHG3_99	95,461-95,814	Fwr.	Hypothetical protein
SPHG3_100	95,922-98,663	Fwr.	rIIA lysis inhibitor
SPHG3_101	98,696-100,279	Fwr.	rIIA lysis inhibitor
SPHG3_102	100,332-100,631	Fwr.	Hypothetical protein
SPHG3_103	100,549-101,019	Fwr.	Hypothetical protein
SPHG3_104	101,052-101,444	Fwr.	Hypothetical protein
SPHG3_105	101,423-102,232	Fwr.	Tail fiber
SPHG3_106	102,506-103,048	Fwr.	Hypothetical protein
SPHG3_107	103,329-103,814	Fwr.	Hypothetical protein
SPHG3_108	103,811-104,389	Fwr.	Hypothetical protein
SPHG3_109	104,439-106,352	Fwr.	DNA topoisomerasse
SPHG3_110	106,354-107,685	Fwr.	DNA Topoisomerase
SPHG3_111	107,729-108,007	Fwr.	Hypothetical protein
SPHG3_112	108,009-108,320	Fwr.	Hypothetical protein
SPHG3_113	108,324-108,737	Fwr.	Hypothetical protein
SPHG3_114	108,787-109,011	Fwr.	Hypothetical protein
SPHG3_115	109,008-109,244	Fwr.	Hypothetical protein
SPHG3_116	109,226-109,846	Fwr.	Hypothetical protein
SPHG3_117	109,843-110,187	Fwr.	Hypothetical protein
SPHG3_118	110,180-110,812	Fwr.	Hypothetical protein
SPHG3_119	110,890-111,180	Fwr.	Exonuclease A
SPHG3_120	111,252-111,548	Fwr.	Hypothetical protein
SPHG3_121	111,548-112,210	Fwr.	Hypothetical protein
SPHG3_122	112,213-112,554	Fwr.	Hypothetical protein
SPHG3_123	112,536-113,003	Fwr.	Hypothetical protein
SPHG3_124	113,003-113,563	Fwr.	putative serine/threonine protein phosphatase
SPHG3_125	113,560-113,892	Fwr.	Hypothetical protein

SPHG3_126	113,889-114,239	Fwr.	Hypothetical protein
SPHG3_127	114,620-115,141	Fwr.	dCMP deaminase
SPHG3_128	115,143-115,550	Fwr.	membrane-flanked domain protein
SPHG3_129	115,760-116,380	Rev.	Head completion protein
SPHG3_130	116,434-117,402	Fwr.	Baseplate tail tube cap
SPHG3_131	117,415-117,972	Fwr.	Baseplate wedge component
SPHG3_132	117,969-119,357	Fwr.	Hypothetical protein
SPHG3_133	119,368-121,308	Fwr.	Hypothetical protein
SPHG3_134	121,309-121,971	Rev.	DNA helicase loader
SPHG3_135	122,456-123,883	Rev.	DNA Ligase
SPHG3_136	127,246-128,670	Rev.	DNA primase/ helicase
SPHG3_137	128,984-130,069	Rev.	Recombination protein
SPHG3_138	130,584-131,135	Rev.	putative dUTP diphosphatase
SPHG3_139	131,132-131,701	Rev.	putative deoxynucleotide monophosphate kinase
SPHG3_140	131,698-132,738	Rev.	Thymidylate synthase
SPHG3_141	135,719-136,423	Rev.	DNA end protector during packaging
SPHG3_142	136,709-137,419	Fwr.	Tail tube monomer
SPHG3_143	137,566-138,477	Rev.	Single stranded DNA-binding protein
SPHG3_144	138,823-139,125	Rev.	Late promoter transcription accessory protein
SPHG3_145	139,126-139,479	Fwr.	Hypothetical protein
SPHG3_146	139,495-139,770	Rev.	Hypothetical protein
SPHG3_147	139,875-140,513	Rev.	Hypothetical protein
SPHG3_148	140,513-141,130	Rev.	Hypothetical protein
SPHG3_149	141,492-141,821	Rev.	Hypothetical protein
SPHG3_150	141,822-143,447	Fwr.	Cell wall-associated hydrolases
SPHG3_151	143,512-143,892	Fwr.	Baseplate wedge subunit
SPHG3_152	145,270-146,373	Rev.	ribonucleotide reductase of class Ia (aerobic) beta subunit
SPHG3_153	146,444-148,771	Rev.	ribonucleotide reductase of class Ia (aerobic) alpha subunit
SPHG3_154	148,892-149,011	Fwr.	peptidoglycan hydrolase
